# An ethical basis for research into health and climate change 

**DOI:** 10.2471/BLT.26.295838

**Published:** 2026-03-01

**Authors:** Katharine Wright, Katherine Littler

**Affiliations:** a London, England.; b Science for Health Department, World Health Organization, Avenue Appia 20, 1211 Geneva 27, Switzerland.

This theme issue of the *Bulletin of the World Health Organization* invited contributions on topics shaping ethically sound climate and health research.^1^ The articles cover a range of methods, populations and disciplines, reflecting the growing importance of the field of ethics in supporting effective and equitable responses to the climate threat to health. Given the breadth of health and climate challenges covered, a striking number of common ethical themes emerge across these diverse contributions, providing a clear indication for where further ethical guidance may be valued.

First is the importance of being explicit about the values that underpin consequential decisions, whether in research itself or in its subsequent impact on policy and practice. Ethical concerns are often not labelled as such, but several articles illustrate how ethical values are built into the assumptions and models that inform decisions of real-life import.^2^^,^^3^ Economic models, for example, use discounting approaches that contain value judgements about the relative importance of impacts on future people, or disguise inequitable impacts by incorporating assumptions that health gains and/or costs will be uniformly experienced. The need for greater ethical explicitness is emphasized through highlighting the role of decision science in exposing these underlying ethical choices to scrutiny.^2^

A second theme is that of the number and diversity of the stakeholders involved in climate and health. In addition to the role of the health-care sector itself, including research actors and supply chains,^4^^,^^5^ contributors highlight multiple sectors of national and local government that exercise climate-related public health responsibilities, from urban planning^6^ to flood defence.^7^ Another article extends this range of duty-bearers to include commercial actors, singling out the role of the insurance industry in helping perpetuate the carbon-intensive industries that worsen the health outcomes they are meant to protect against.^8^ This complex combination of responsibilities borne by public and private entities raises several ethical issues, both theoretical and practical. Two articles address the justification for allocating such responsibilities, including with reference to constitutive duties, culpability, past and ongoing benefit and ability to act.^9^^,^^10^ A distinction is then proposed between the enabling responsibilities of higher-income countries to support adaptation through funding and infrastructure strengthening, and enacting responsibilities that should rest with the governments of lower-income countries based on their agency and local knowledge.^10^ This distinction makes a powerful case for an ethical distribution of responsibilities that supports locally led, relevant adaptation research while avoiding both abandonment and domination.

The fulfilment of intersecting responsibilities requires effective and respectful collaboration between the many agencies implicated, an aspiration that can only be realized if actions are underpinned by mutual respect for different forms of knowledge, coupled with a willingness to listen, learn and adapt. A degree of humility and a strong focus on epistemic justice are required. These ethical concepts have enormous practical resonance. Almost all articles reiterate the importance of giving due weight to contextual knowledge and lived experience if research is to offer real prospect of benefiting those most affected by climate change. In particular, authors presenting case studies in Viet Nam and Zimbabwe argue for research to incorporate renegotiated power relations, address existing structural inequities, and ensure that outcomes translate into timely and tangible benefits.^11^

Finally, this theme issue includes substantive contributions in two areas of ethical interest and contestation in climate and health research. First, how concern for intergenerational justice can in practice be incorporated into research and policy decisions.^8^^,^^9^^,^^12^ Second, whether intrinsic value is to be placed on the well-being of the environment and non-human animals.^9^^,^^12^ Calls are made for any discounting of future benefit to be explicitly justified;^2^ for the interests of people living in the distant future to be given similar weight as those alive now in priority-setting mechanisms for climate–health research;^9^ and for more sustainable practices across all sectors to be embraced as a necessary part of protecting the interests of future generations.^4^^,^^5^^,^^8^ While such arguments weigh environmental interests instrumentally in service to human health, other contributors argue for those interests to be given some independent weight. In research priority-setting, an article proposes that environmental benefits be given similar, but perhaps not equal, weight relative to human health benefits,^9^ while another article explores whether the remit of research ethics committees could be extended to include non-human health concerns, with particular reference to African environmental ethical traditions that lean towards ecocentrism.^12^

This theme issue highlights the value that ethical analysis can bring in achieving equitable climate and health action. This important area of research is a growing one, and we hope this issue is the start of an ongoing conversation.
